# Transcriptomic Analysis Reveals the Regulatory Networks and Hub Genes Controlling the Unsaturated Fatty Acid Contents of Developing Seed in Soybean

**DOI:** 10.3389/fpls.2022.876371

**Published:** 2022-05-12

**Authors:** Junqi Liu, Liang Dong, Runqing Duan, Li Hu, Yinyue Zhao, Liang Zhang, Xianzhi Wang

**Affiliations:** ^1^School of Agriculture, Yunnan University, Kunming, China; ^2^Food Crops Research Institute, Yunnan Academy of Agricultural Sciences, Kunming, China

**Keywords:** soybean, unsaturated fatty acids, developing seeds, RNA-seq, regulatory network, WGCNA

## Abstract

Soybean [*Glycine max* (L.) Merr.] is one of the most important crops, which produces about 25% of the world’s edible oil. The nutritional value of soybean oil depends mostly on the relative contents of three unsaturated fatty acids (UFAs), i.e., oleic acid, linoleic acid (LA), and linolenic acid. However, the biosynthetic mechanism of UFAs remains largely unknown, and there are few studies on RNA-seq analysis of developing seeds. To identify the candidate genes and related pathways involved in the regulation of UFA contents during seed development in soybean, two soybean lines with different UFA profiles were selected from 314 cultivars and landraces originated from Southern China, and RNA-seq analysis was performed in soybean seeds at three developmental stages. Using Gene Ontology (GO) and Kyoto Encyclopedia of Genes and Genomes (KEGG) enrichment analysis, a series of genes and pathways related to fatty acid metabolism were identified, and 40 days after flowering (DAF) was found to be the crucial period in the formation of UFA profiles. Further, weighted gene co-expression network analysis identified three modules with six genes whose functions were highly associated with the contents of oleic and LA. The detailed functional investigation of the networks and hub genes could further improve the understanding of the underlying molecular mechanism of UFA contents and might provide some ideas for the improvement in fatty acids profiles in soybean.

## Introduction

Soybean [*Glycine max* (L.) Merr.] is one of the most important oil crops in the world, which produces about 25% of the world’s edible oil (Maranna et al., 2021). Soybean oil is not only an essential food supply but also has industrial and energy production use. The nutrition and utilization values of soybean oil depend on the seed fatty acid composition (Ensminger and Ensminger, 1993).

Soybean seeds consist predominantly of five fatty acids: palmitic acid (16:0, PA), stearic acid (18:0, SA), oleic acid (18:1, OA), linoleic acid (18:2, LA), and linolenic acid (18:3, LNA) (Wilson, 1998). PA and SA are the saturated fatty acids which are stable to the oxidation process, but too much intake of them may cause problems such as heart cerebrovascular disease and prostate cancer (Hu et al., 1997). LA and LNA are the polyunsaturated fatty acids (PUFAs) which are oxidized easily (Yadav et al., 1993; Warner and Gupta, 2005). OA is the monounsaturated fatty acid which has good autoxidative stability (Okeefe et al., 1993) and can reduce the risk of cardiovascular diseases and suppresser tumorigenesis of inflammatory diseases (Yamaki et al., 2005; Sales-Campos et al., 2013). Therefore, soybean oil with a high percentage of OA is preferable from a nutritional and technological point of view. Developing soybean cultivars with high OA and low PUFAs to improve the edible soybean oil quality has been a long-time goal for soybean breeders (Clemente and Cahoon, 2009; Kanai et al., 2019).

In the recent decades, genes involved in fatty acid metabolism have been studied extensively; e.g., β-ketoacyl-acyl carrier protein synthase (KASI) catalyzes the elongation of *de novo* fatty acid synthesis and changes the fatty acid compositions and contents in soybean seeds (Wu and Xue, 2010; Ding et al., 2015; Dobbels et al., 2017), overexpression of the acetyl CoA carboxylase (ACCase) in the amyloplasts of potato can increase the amount of triacylglycerol for fivefolds (Klaus et al., 2004), fatty acid desaturase 2 (FAD2) deficiency results in a remarkable change in the contents of unsaturated fatty acid (UFAs) (Haun et al., 2014; Do et al., 2019), and triacylglycerols lipase preferentially cleaves OA than LA in the oil body membrane, thus regulating the oleic or LA ratio in soybean seeds (Kanai et al., 2019). Besides, there are some key transcription factors (TFs) in the regulation of lipid biosynthesis including WRI1 (Ma et al., 2015), LEC1 (Tan et al., 2011), LEC2 (Baud et al., 2007), FUS3 (Wang et al., 2007), and ABI3 (Crowe et al., 2000). As mentioned above, considerable progresses have been achieved for the study of genes involved in plant fatty acids metabolism. However, an analysis of the soybean genome identified over 250 gene homologs governing soybean seed oil as well as fatty acid storage and metabolism (Schmutz et al., 2010), indicating that there is still a huge unknown in the regulatory network.

Currently, the study of molecular mechanisms for the formation of crop traits has been promoted by “-omics” approaches, such as RNA-seq (Yuan et al., 2021). For example, RNA-seq was conducted at the developing seeds of three peanuts and identified some candidate genes responsible for the seed size or weight in peanut (Li et al., 2021). In rapeseed, comparative transcriptome analysis was carried out on two accessions of R- (resistant in clubroot) and S- (susceptible in clubroot) lines. The result revealed some pathways involved in the regulation of clubroot resistance and 12 hub genes (Li et al., 2020). But in soybean, transcriptome analysis of seeds at different developmental stages is not widely reported (Asakura et al., 2012; Lu et al., 2016; Du et al., 2017). In this study, two soybean landraces with stable differences in UFA profiles through three environments were selected from a screen of 314 cultivars and landraces originated from Southern China. Soybean seeds at three developmental periods were chosen for RNA-seq to uncover the mechanism of these differences at the transcriptional level. We expect that this research could enrich the understanding of the regulatory network of UFA metabolism in soybean seeds and could provide a theoretical reference for breeding soybean with preferable fatty acid profiles.

## Materials and Methods

### Plant Materials

A soybean panel consisting of 314 cultivars and landraces originated from Southern China was planted in a randomized complete block design with two replications under three environments, i.e., Anning, Kunming, Yunnan, China, 2019 and 2020, and Menghai, Xishuangbanna, Yunnan, China, 2020. All the plots were bulk harvested individually after full maturity, and the fatty acid profiles of the seeds were determined using a near-infrared analyzer. A total of two landraces originated from Yunnan Province with stable different seed fatty acid profiles through three environments were selected and used as the plant materials for further analysis. The line with high OA and low PUFAs (Chinese Crop Germplasm Information System, accession number ZDD17348, namely, HO) and the line with low oleic and high PUFAs (Chinese Crop Germplasm Information System, accession number ZDD17370, namely, LO) were planted in the greenhouse in 2021 for sample collection in the Agricultural Station of Yunnan University in Kunming, China. The two groups of soybean seed samples from HO and LO were collected every 10 days starting from 20 to 70 days after flowering (DAF), respectively. A number of three biological replicates were used for each of the sampling points. Thus, 36 samples, namely, LO 20 a-c, LO 30 a-c, LO 40 a-c, LO 50 a-c, LO 60 a-c, LO 70 a-c, HO 20 a-c, HO 30 a-c, HO 40 a-c, HO 50 a-c, HO 60 a-c, and HO 70 a-c were collected. The samples were quickly frozen in liquid nitrogen and stored at −80°C for further analysis.

### Determination of Fatty Acids by Gas Chromatography

About 30 mg soybean seeds from each sample were used for methyl esterification and then filtered into a gas chromatographic bottle for gas chromatography (GC) detection. Each sample was measured 3 times, the GC program was as follows: injection volume of 1 μl, split 10:1, starting temperature at 90°C, heating rate at 20°C/min to 160°C, holding for 1 min, then heating rate at 2°C/min to 220°C, holding for 2 min; helium: 40 ml/min, hydrogen: 35 ml/min, air: 350 ml/min; injection port temperature: 250°C, flame ion detector: 250°C.

### RNA-Seq and Data Preprocessing

Soybean seeds at 30, 40, and 50 DAF were collected for three replications at each stage. Total RNAs were extracted from each sample and tested for quality, i.e., the purity, concentration, and integrity and then sent out for RNA-seq. The sequencing was performed using Illumina high-throughput sequencing platform based on sequencing by synthesis technology by Biomarker Technologies Company, Beijing, China. The clean reads were mapped to the reference genome of soybean Glycine_max_v4.0 by HISAT2 (Kim et al., 2015), and StringTie was used to assemble and quantify (Pertea et al., 2015). Fragments per kilobase of exon model per million mapped fragments (FPKM) was used to measure the transcription or gene expression level (Florea et al., 2013). Pearson correlation coefficient analysis was performed with the R packages corrplot^1^ to evaluate reproducibility between samples.

### Validation of RNA-Seq Data

The quantitative real time polymerase chain reaction (qRT-PCR) was used to determine the relative expression levels of six differentially expressed genes (DEGs) at different periods and to verify the RNA-seq results. Gene-specific primers for qRT-PCR are listed in Supplementary Table 2, and soybean gene *TUBB3* (β-tubulin, NCBI Gene ID: 547844) was used as internal standard. Each reaction was performed in three technical replicates. The relative expression fold changes of genes in HO versus LO were analyzed using the 2^–Δ^ ^Δ^ *^Ct^* method (Livak and Schmittgen, 2001). The fold changes of genes in HO versus LO obtained *via* RNA-seq were calculated *via* FPKM. The log_2_ fold change values of quantitative polymerase chain reaction (qPCR) and RNA-seq of DEGs were used for graphical presentation.

### Analysis of Differential Gene Expression and Gene Annotation

Differential expression analysis was performed using the DEseq2 R package. DEGs were filtered with | log_2_ fold change| ≥ 1 and false discovery rate (FDR) < 0.01. All expressed genes were functionally annotated using Gene Ontology (GO) database and Kyoto Encyclopedia of Genes and Genomes (KEGG) database. The GO terms and the KEGG pathways with an *q*-value < 0.05 were defined as significantly enriched terms or pathways.

### Weighted Gene Co-expression Network Analysis and Visualization

Weighted gene co-expression network analysis (WGCNA) was performed according to Langfelder and Horvath (2008). Gene cluster dendrogram was constructed with a power value = 15. The gene modules were further classified and clustered by similarity = 0.8, and minModuleSize = 30. The top 100 pairs of genes with the highest weighted value in each module were selected for co-expression network construction, genes with high weight values were defined as hub gene, and the network of traits specific modules was visualization using Cytoscape3.9.0.

## Results

### HO and LO Plant Material Selection and Fatty Acid Profile Analysis

**FIGURE 1:**
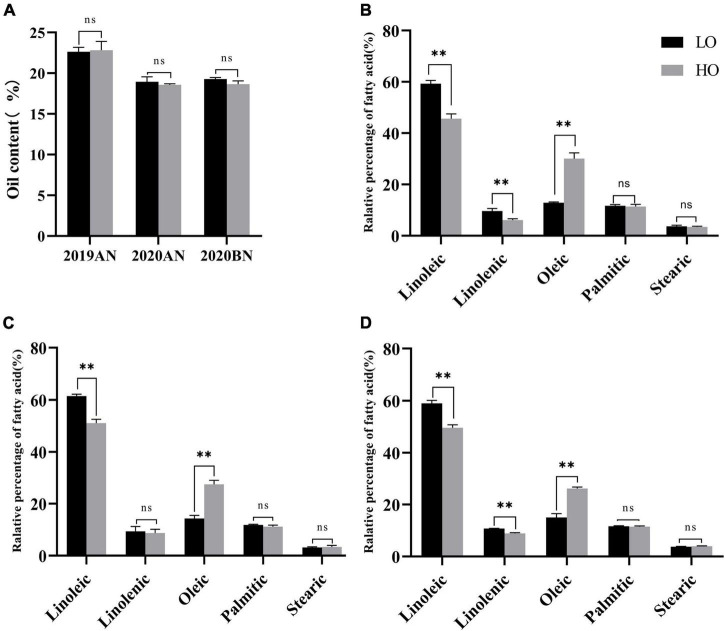
Comparison of fatty acid content and fatty acid profiles between HO and LO. *T*-test was performed between the paired samples under different environments. ^**^ and ns represent significant at *P* ≤ 0.01 and none significant, respectively. **(A)** Comparison of total fatty acid contents in 2019AN, 2020AN and 2020BN; **(B)** Comparison of fatty acid profiles in 2019AN; **(C)** Comparison of fatty acid profiles in 2020AN **(D)** Comparison of fatty acid profiles in 2020BN. 2019AN, 2020AN, and 2020BN represent the environment of Anning (2019, 2020), and Xishuangbanna (2020), respectively.

Among 314 cultivars and landraces originated from Southern China, two landraces with stable differences in UFA profiles through three environments were selected, namely, HO and LO. There was no significant difference in total fatty acid content between the two lines (Figure 1A), but significant differences in UFA profiles (Figures 1B–D).

**FIGURE 2:**
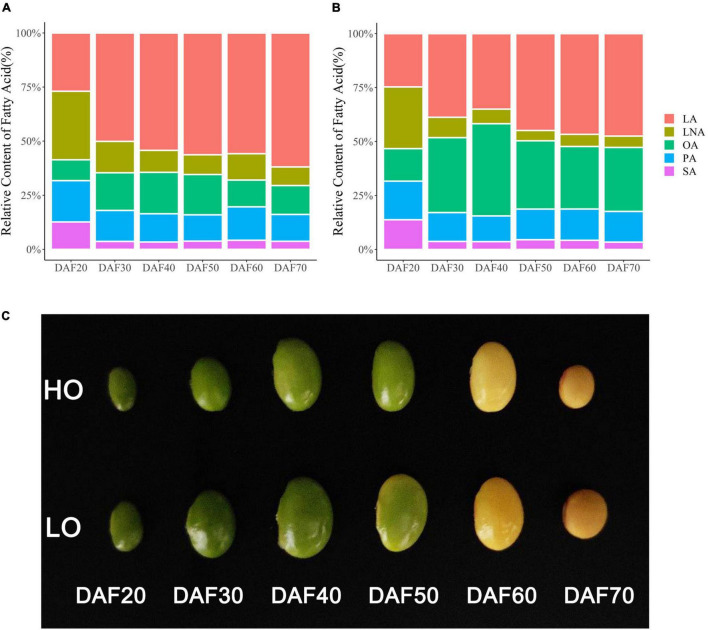
Fatty acid profiles of two materials at different periods. **(A)** Fatty acid profiles of LO at different periods. **(B)** Fatty acid profiles of HO at different periods. **(C)** Soybean seeds at different development stages. PA, palmitic acid; SA, stearic acid; OA, oleic acid; LA, linoleic acid; LNA, linolenic acid.

In LO seeds, the relative content of LA increased continuously through the seed development and became dominant at 30 DAF, with the final content of about 55%. The relative content of OA reached the maximum at 40 DAF and then decreased to about 13%. The relative content of LNA was about 32% at 20 DAF and then gradually decreased to 8% (Figure 2A). In HO seeds, the relative content of OA increased continuously, reached its maximum value at 40 DAF, and then decreased at 50 DAF, with the final content of about 30%. The relative content of LA increased continuously, with the final content of about 46%. Additionally, the relative content of LNA was about 29% at 20 DAF and then decreased at 30 DAF, with the final content of about 5% (Figure 2B). As the relative contents of UFAs of two soybeans began to differ obviously at 30 DAF, the difference was the largest at 40 DAF and then decreased at 50 DAF (Figures 2A,B); therefore, soybean seeds at 30, 40, and 50 DAF were selected for transcriptome analysis. Besides the relative UFA contents, there were also significant differences in the seed size between HO and LO through the seed developmental stages (Figure 2C).

### Analysis of Transcriptome Data

Soybean seeds at 30, 40, and 50 DAF were sampled from LO and HO, respectively. In total, 18 libraries were constructed and analyzed, and 134.10 Gb clean data were obtained. The percentage of Q30 (quality value larger than 99.9%) base was over 92.83%, and 88.48–96.56% of reads were mapped to soybean genome uniquely in each sample. Genes with normalized reads lower than 0.5 FPKM were removed from the analysis. In total, 27,795, 27,209, and 22,991 transcripts were found to be expressed in 30, 40, and 50 DAF in LO, respectively. Similarly, 27,135, 34,800, and 23,638 transcripts were identified in the correspondent samples in HO, respectively. Approximately 74% of expressed genes were in the range of 0.5–10 FPKM, 24% of expressed gene were in the range of 10–100 FPKM, and less than 2% of expressed gene were more than the range of 100 FPKM (Supplementary Figure 1). The overlaps of expressed genes in the three samples of HO and LO are shown in Supplementary Figure 1. To evaluate the reliability of different biological replicates, correlation analysis was performed among different replicates (Supplementary Figure 1). The result showed that there were significant correlations among different replicates, showing the high reliability of the RNA-seq data.

### Identification of Differentially Expressed Genes and Functional Annotation

To identify the DEGs, we conducted a pairwise comparison at each developmental stage between HO and LO. Differential expression analysis showed that 2,080 genes at 30 DAF, 11,343 genes at 40 DAF, and 2,230 genes at 50 DAF were found to be differentially expressed between HO and LO (| log_2_ fold change| ≥ 1 and FDR < 0.01).

**FIGURE 3:**
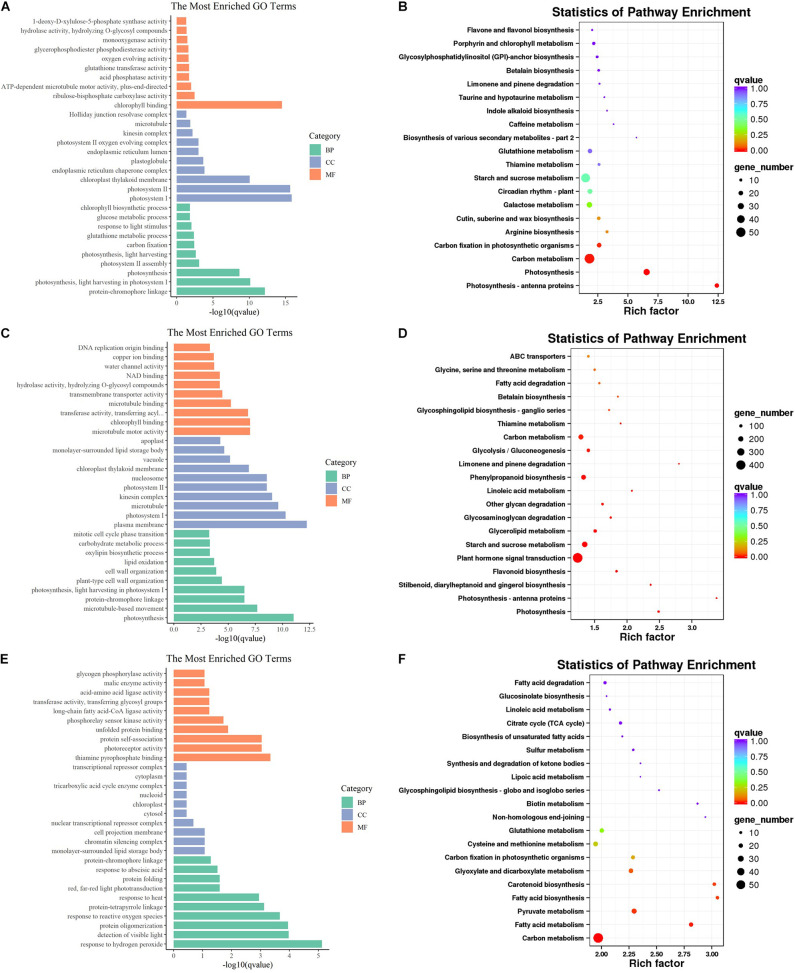
Gene Ontology and KEGG enrichment analysis of DEGs. The most enriched GO terms of DAF 30 **(A)**, DAF40 **(C)** and DAF 50 **(E)**. Top 20 KEGG pathways of DAF 30 **(B)**, DAF 40 **(D)** and DAF50 **(F)**.

To preliminarily explore the function of these DEGs, GO analysis was conducted and many GO terms were significantly enriched (*q* < 0.05). These GO terms belong to three categories: biological process (BP), cellular component (CC), and molecular function (MF). For the BP ontology, the representational enriched terms were “protein-chromophore linkage,” “photosynthesis, light harvesting in photosystem I,” “photosynthesis,” and “response to hydrogen peroxide” (Figures 3A,C,E). For the CC ontology, the representational enriched terms were “photosystem I,” “photosystem II,” “plasma membrane,” and “monolayer-surrounded lipid storage body” (Figures 3A,C,E). For the MF ontology, the representational enriched terms were “chlorophyll binding,” “microtubule motor activity,” and “thiamine pyrophosphate binding” (Figures 3A,C,E). Subsequently, the DEGs were mapped to the reference canonical pathways in the KEGG database. According to the KEGG annotations, 25 pathways were significantly enriched (*q* < 0.05). “Photosynthesis – antenna proteins,” “Photosynthesis,” “Carbon metabolism,” and “Carbon fixation in photosynthetic organisms” were significantly enriched at 30 DAF. “Photosynthesis – antenna proteins,” “Photosynthesis,” “Flavonoid biosynthesis,” “Starch and sucrose metabolism,” and “Linoleic acid metabolism” were significantly enriched at 40 DAF. “Carbon metabolism,” “Fatty acid metabolism,” “Pyruvate metabolism,” “Fatty acid biosynthesis,” and “Carotenoid biosynthesis” were significantly enriched at 50 DAF.

**FIGURE 4:**
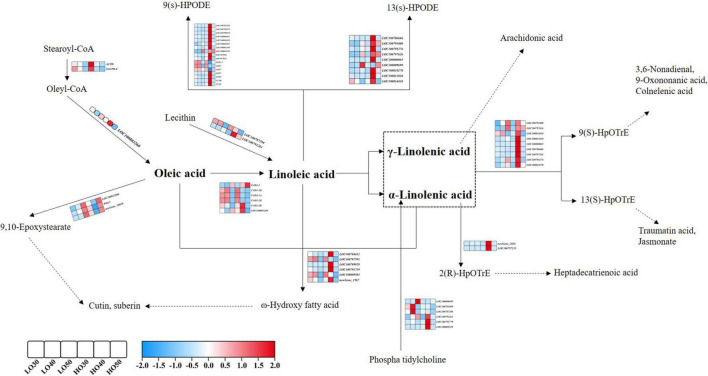
Overview of the distribution of DEGs in the metabolic pathway of oleic and linoleic acids during soybean seed development. Gene IDs or names and expression patterns are indicated. The expression pattern of each gene is shown by 6 grids, which representing the relative log2 (expression ratio) at LO 30, LO 40, LO 50, HO 30, HO 40, and HO 50, respectively.

Notably, there were some pathways directly related to fatty acid profiles at different stages. “Cutin, suberin, and wax biosynthesis” was enriched at both 30 and 40 DAF. “Fatty acid metabolism” was significantly enriched at 50 DAF. “Fatty acid elongation” and “Linoleic acid metabolism” were significantly enriched at 40 DAF. Therefore, we focused on the analysis of these fatty acid-related pathways (fatty acid metabolism, fatty acid degradation, fatty acid elongation, LA metabolism and cutin, suberin, and wax biosynthesis). A large number of genes that affect fatty acid profiles were differentially expressed when comparing HO with LO at different stages. For example, the FAD2-encoding genes, *FAD2-1A* and *FAD2-1B*, were significantly downregulated from LO to HO, some lipoxygenase-encoding genes were upregulated from LO to HO, and many genes whose functions remain unclear were also differentially expressed between HO and LO. Interestingly, many of these genes were specifically high or low expressed in HO 40 (Figure 4).

### Weighted Gene Co-expression Network Analysis and Visualization

**FIGURE 5:**
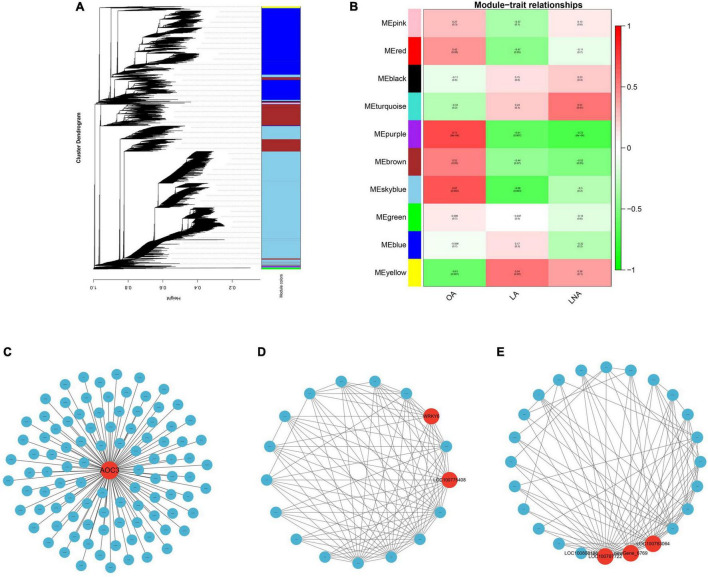
Weighted gene co-expression network analysis and visualization. **(A)** Hierarchical cluster tree showing co-expression modules identified by WGCNA. Each leaf (short vertical line) in the tree represented one gene. The major tree branches constitute 10 modules labeled by different colors. **(B)** Module-trait relationships. Each row corresponded to a module, and each column corresponded to a trait. OA, oleic acid; LA, linoleic acid; LNA, linolenic acid. The correlation network of genes in skyblue **(C)**, purple **(D)** and yellow **(E)** modules. Red circle indicated hub genes.

Weighted gene co-expression network analysis was performed using 27,831 genes that had an average FPKM >1 in all samples. Based on the correlation coefficients of these genes, a gene cluster dendrogram was constructed and classified. Modules were defined as clusters of highly interconnected genes, and genes within the same cluster have highly correlation coefficients among them. Finally, 10 distinct modules (labeled with different colors) were identified (Figures 5A,B). To identify trait-related modules, the correlation coefficient between modules and traits (the relative content of UFAs) was calculated. As a result, 3 out of 10 co-expression modules (yellow, purple, and sky blue) showed a significant association with UFA content changes during soybean seed development (Figure 5). The yellow module with 141 identified genes showed a positive correlation with LA and negative correlation with OA, whereas the purple module (representing 139 genes) and sky blue module (representing 13,646 genes) showed a positive correlation with OA, but negative correlation with LA and LNA. The genes of sky blue module were highly expressed in HO 40 (Supplementary Figure 2), which has the highest oleic/PUFA ratio among all samples (Figure 2). While the genes of the purple module were highly expressed in LO 50 (Supplementary Figure 2), which has the lowest oleic/PUFA ratio (Figure 2), the genes of the yellow module showed a clearly different expression pattern between LO and HO. The other modules also exhibited a correlation with UFAs, although no significant difference was observed.

Gene Ontology term enrichment analysis for three significantly correlated modules was conducted (*q* < 0.05). According to the MF, sky blue module was mainly enriched in chlorophyll binding, protein heterodimerization activity, and structural constituent of ribosome. Purple module was mainly enriched in phosphatidylinositol-4-phosphate binding and ribonucleoside-diphosphate reductase activity. The genes in the yellow module were not significantly enriched to any terms. According to CC, sky blue module was mainly enriched in Golgi apparatus, Golgi membrane, and nucleosome. Purple module was mainly enriched in endoplasmic reticulum (ER) component, such as ER membrane and ER subcompartment. The genes in the yellow module were not significantly enriched to any terms. In BPs, sky blue module was significantly enriched in intracellular protein transport, translation, and photosynthesis. Purple module was mainly enriched in the regulation of leaf senescence and regulation of leaf development. Yellow module significantly enriched in fructose 2,6-bisphosphate metabolic process.

In KEGG pathway enrichment analysis (Supplementary Table 5), sky blue module was represented by ribosome, biosynthesis of amino acid, carbon metabolism, oxidative phosphorylation, phagosome, and so on (*q* < 0.05). Meanwhile, cutin, suberin, and wax biosynthesis, fatty acid degradation, fatty acid elongation, fatty acid metabolism, and LA metabolism also enriched in brown module, although no significant difference was observed. In addition, there were no significant enrichment pathways in purple and yellow modules (*q* < 0.05).

The correlation network of the sky blue module is shown in Figure 5C. *AOC3* was identified as the candidate hub genes, and this gene encodes a vascular adhesion protein 1. In the purple module, a TF-encoding gene (*WRKY6*) and a pentatricopeptide repeat-containing protein-encoding gene (*LOC100775408*) were identified as the candidate hub genes (Figure 5D). Tubulin alpha-5 chain-encoding gene (*LOC100783064*), calcineurin-binding protein-encoding gene (*LOC100787722*), and an unknown function gene were identified as the candidate hub genes of the yellow module (Figure 5E).

### The Validation of RNA-Seq Data

**FIGURE 6:**
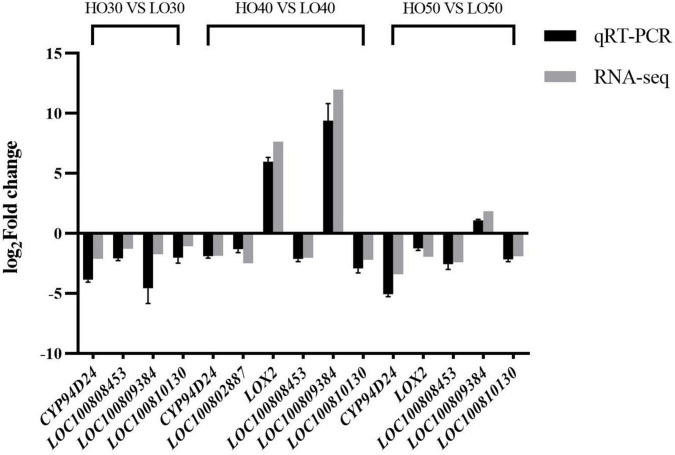
The quantitative real time polymerase chain reaction verification of six genes related to fatty acid metabolism.

To verify the quality of the RNA-seq and differential expression level data, qRT-PCR was used to detect the expression levels of six fatty acid metabolism-related genes in three periods. The changes in the expression of selected genes according to qRT-PCR showed a similar expression tendency to the RNA-seq data (Figure 6), indicating that transcriptomic profiling data were highly reliable.

## Discussion

Currently, the nutritional quality of food is a growing concern. As one of the main edible oils, the nutritional value of soybean oil depends mostly on fatty acid profiles of seeds, especially the ratio of OA, LA, and LNA. Understanding the molecular mechanisms controlling fatty acid profiles is of great importance for the improvement in the nutritional value of soybean oil. In addition, there are a large number of soybean landraces that contain many excellent genetic variations, but are not fully utilized. The objectives of this study were to screen elite germplasm with preferable UFA profile originated from Southwest China and to identify candidate genes and gene expression network controlling UFA contents during the development of seeds in soybean.

The two soybean landraces, HO (with stable high oleic/PUFA ratio) and LO (with stable low oleic/PUFA ratio), were screened out from 314 landraces and cultivars. To determine the period of RNA-seq sampling, seeds were collected during fatty acid accumulation (Qi et al., 2018; Ma et al., 2021), and fatty acid profiles of two landraces at different seed developmental stages were determined by gas chromatograph. Phenotypic analysis showed that there were significant differences for UFA contents between the two lines, and the largest difference was at 40 DAF (Figure 2), indicating that 40 DAF might be the most vigorous period of soybean seed metabolism. Transcriptomic analysis identified a number of genes which were differentially expressed in developing soybean seeds between two landraces. About 74% of expressed genes were in the range of 0.5–10 FPKM, and only a small fraction of expressed genes (less than 2%) was expressed at relatively high levels (100 ≤ FPKM). The number of expressed genes in both lines was highest at 40 DAF, further indicating that 40 DAF might be the most vigorous period of soybean seed metabolism. Besides, the expression profile of LO 30 was highly correlated with LO 40, which contrasted with HO 30 and HO 40 (Supplementary Figure 1). This might be due to the different growth velocities at the stage of 30 and 40 DAF between LO and HO lines. LO line developed slowly at 30–40 DAF, which might cause similar gene expression profiles at 30 and 40 DAF and might result in high correlation between LO 30 and LO 40.

Gene Ontology term analysis showed that the DEGs of HO and LO were significantly enriched in the GO terms (such as photosynthesis, chloroplast thylakoid membrane, and chlorophyll binding, etc.), which are related to photosynthesis and chloroplast. Acetyl-CoA is an important substance for the *de novo* synthesis of fatty acids (Slabas and Fawcett, 1992), and photosynthesis in chloroplasts can provide endogenous carbon sources for the formation of acetyl-CoA, thus affecting fatty acid metabolism (Arent et al., 2008). Soybean seeds are photosynthetic seeds, whose formation of fatty acids can be affected through photosynthesis (Allen et al., 2009; Houston et al., 2009). Therefore, in the early and middle stages of growth and development, HO and LO may differ in the accumulation of fatty acids due to the differences in photosynthetic efficiency and utilization of photosynthates. The synthesis and transport of fatty acids involve plasmid membrane, ER, and plasma membrane (Benning, 2009). After synthesis and processing in the cell, most fatty acids are transported to the extracellular domain through the plasma membrane, secreted by lipid transfer proteins (LTPs), and participate in the assembly of cutin, suberin, and sporopollenin (DeBono et al., 2009; Edstam et al., 2013). Many studies have shown that LTPs preferentially bind with certain fatty acids (Sawano et al., 2008), and knockout or overexpression of *LTPs* can affect the composition of plant fatty acids (Liu et al., 2014; Deeken et al., 2017; Lee and Suh, 2018). In this research, the DEGs of 40 DAF was quite significantly enriched in “plasma membrane” (Figure 3), and the expression levels of LTP genes of this term were higher in HO 40 than in LO 40 (Supplementary Table 6). It is speculated that these proteins may preferentially transport LA to extracellular metabolism, resulting in the difference of LA content between the two landraces. In addition, the oleosins are also closely related to fatty acids, and overexpressing or knockout oleosin-encoding gene can affect the fatty acid profiles of plant seeds (Siloto et al., 2006; Chen et al., 2019). There were 12 oleosin-encoding genes significantly enriched in “monolayer-surrounded lipid storage body” at 40 and 50 DAF (Supplementary Table 6), indicating that oleosin-encoding genes might contribute to fatty acid profiles in soybean.

Based on the gene differential expression analysis and KEGG database, the distribution map of DEGs in the metabolic pathways of UFAs was preliminarily constructed. Interestingly, most of these genes were specifically high or low expressed in HO 40, and the DEGs between HO 40 and LO 40 are enriched in many fatty acid-related pathways. As shown in Figure 4, *ACPD* and *SACPD-C* were highly expressed in HO 30, which encode a stearoyl-acyl carrier protein desaturase and a stearoyl-ACP desaturase-converting stearoyl-CoA to oleyl-CoA (Kachroo et al., 2007; Gillman et al., 2014). Another gene, *LOC100803260*, was also highly expressed in HO 40, which encode protein-converting oleyl-CoA to OA. The FAD2-encoding genes, *FAD2-1A* and *FAD2-1B*, which are the key genes that convert OA to LA (Pham et al., 2011), were significantly downregulated from LO to HO. This might be helpful for the accumulation of high OA in HO. Notably, other FAD2-encoding genes such as *FAD2-2* were upregulated from LO to HO, which contradicts the accumulation of OA content. In a previous study, the effect of *FAD2-2* on OA content was proved to be lower than those of *FAD2-1A* and *FAD2-1B* (Al Amin et al., 2019; Do et al., 2019). In this study, the upregulation of *FAD2-2* from LO to HO could not counteract the effects of *FAD2-1A* and *FAD2-1B*. This might due to the relative smaller effect of *FAD2-2*, and the fold change of *FAD2-2* between HO and LO was much lower than those in *FAD2-1A* and *FAD2-1B* (Supplementary Table 1).

In addition, the utilization of UFAs was different between HO and LO; for example, some DEGs were significantly enriched in cutin, suberin, and wax biosynthesis in this study. Cutin and suberin are the extracellular lipids, which locate on the surface of plants and provide a protective barrier against the evaporation of water and invasion of bacteria (Beisson et al., 2007). OA is the main precursors of cutin and suberin, and it can form C18 cutin monomers with the action of peroxygenase (Blee and Schuber, 1993; Lequeu et al., 2003). In this study, two peroxygenase-related genes (*PM13* and *LOC100913066*) and an uncharacterized gene (*LOC100799842*) were annotated to this pathway. The expression levels of these three DEGs in 40 DAF were lower in HO than in LO, indicating that OA might be used in the formation of cutin and suberin and thus result in the difference in OA content between HO and LO. This might also lead to the differences in water use efficiency and disease resistance between LO and HO, which needs further investigation. In addition, other DEGs in 40 DAF were significantly enriched in LA metabolism. LA is the precursor of plant oxylipins, which forms reactive hydroperoxides in response to lipoxygenases (Vellosillo et al., 2007). Many lipoxygenases-encoding genes were identified to be differentially expressed, such as *LOX7*, *LOX9*, *VLXB*, and *VLXC*. These genes were highly expressed in HO 40, indicating that LA of HO 40 was widely used in the biosynthesis of plant oxylipins, resulting in a decrease in LA content. In addition, a group of cytochrome P450 family genes was also annotated into these pathways. Cytochrome P450 family genes are involved in a variety of biochemical pathways for the production of a range of metabolites and hormones (Schuler and Werck-Reichhart, 2003; Pinot and Beisson, 2011). Cytochrome P450 family members have been recognized to influence fatty acid content of crops. There are four different plant cytochrome P450 subfamilies that catalyze fatty acid ω-hydroxylation, namely, 86A, 86B, 94A, and 704B, but only a few members have been functionally characterized (Bjelica et al., 2016). For example, *CYP86A1* catalyzes ω-hydroxylation of fatty acids to form the ω-functionalized monomers, and knockdown of *CYP86A1* in *Arabidopsis* results in a significant reduction in the content of certain fatty acids (Hofer et al., 2008). *CYP704B1* was found to be needed in Arabidopsis and rice to biosynthesize precursors of sporopollenin thorough oxidizing fatty acids, and overexpression of *PgCYP704B1* in *Arabidopsis* significantly altered the composition of fatty acids in seeds (Silva et al., 2020). *FATB1B* encodes acyl-acyl carrier protein, which terminates the intraplastidial fatty acid synthesis in plants by hydrolyzing the acyl-ACP intermediates and releasing free fatty acids to be incorporated into glycerolipids (Sanchez-Garcia et al., 2010). The mutations at *GmFATB* presented low PA and high OA phenotypes (Zhou et al., 2021). *SACPD* encodes stearoyl-acyl carrier protein, which is essential for the production of the major UFAs in plant lipids. Knocking down the expression of *SACPD* will result in significantly reduced accumulation of 18C UFAs and elevated levels of 18:0-fatty acid (Zhang et al., 2014). Acyl-CoA oxidases catalyze the first step in fatty acid β-oxidation, which breaks down fatty acids by oxidizing the β-carbon atom and removing a two-carbon unit (Arent et al., 2008). In addition, there were many genes specifically expressed at 40 DAF, whose function is yet to be determined, but has the value for further study. Besides, some metabolites derived from PUFAs, such as jasmonates, are beneficial to disease and stress resistance in plants (Heitz et al., 2019). Hence, the balance between UFA content and resistance might be considered during soybean breeding.

Weighted gene co-expression network analysis has been used as a powerful tool in systematic biology for the identification of key genetic networks involved in many crops (Xu et al., 2020; Geng et al., 2021; Xiao et al., 2021). In this study, WGCNA revealed three modules, i.e., sky blue, yellow, and purple module, to be significantly correlated with UFA content in soybean. The genes of these modules were expressed specifically at different periods, suggesting that these three modules may play the important roles in the developing seeds of different stages. Sky blue module was identified as HO 40 stage-specific modules. A vascular adhesion protein 1-encoding gene, *AOC3*, was identified as the hub gene of this module, and this gene is involved in alpha-LNA metabolism. In addition, many fatty acid metabolism-related genes were among the brown module genes, which also showed up in the results of GO and KEGG, indicating that the difference in oleic/PUFA ratio between the two landraces at 40 DAF may depend on a regulatory network composed of these genes. Purple module was a LO 50 stage-specific modules. In this module, a TF-encoding gene, *WRKY6*, and a pentatricopeptide repeat-containing protein-encoding gene, *LOC100775408*, were identified as the candidate hub genes. The relationship between *LOC100775408* and fatty acid metabolism is unclear, whereas the WRKY protein family consists of plant-specific TFs with 182 members, many of which were reported to have multiple functions during the whole plant life cycle in soybean (Bencke-Malato et al., 2014). *WRKY6* was reported to mediate fatty acid profiles through regulating the expression of several genes related to photosynthesis and fatty acid biosynthesis in *Arabidopsis* (Song et al., 2020). However, there is no relevant report in soybean, indicating that these two genes might have potential value for further research. The genes of yellow module showed a clearly different expression pattern between LO and HO at all stages. Among these, a tubulin alpha-5 chain-encoding genes and a calcineurin-binding protein-encoding genes were identified as the candidate hub genes. Tubulin is a component of plant cytoskeleton and participates in material transport and signal transduction in plants (Paradez et al., 2006), whereas calcineurin acts as a crucial connection between calcium signaling the phosphorylation states of numerous important substrates (Creamer, 2020). These two genes may affect some signaling pathways to participate in the regulation of fatty acid metabolism, but no relevant report has been reported in soybean so far. Interestingly, *LOC100808188* was linked to the two hub genes in the co-expression network of yellow module in this study. According to the function prediction, this gene encodes a FATTY ACID EXPORT 3 (FAX3) protein in chloroplast. According to a previous research, *FAX1* was involved in free fatty acids that export from plastids and influence the fatty acid profiles in *Arabidopsis* (Li et al., 2015). Since *FAX3* have high sequence similarity with *FAX1*, we assume that *FAX3* might also have the similar functions, which need further research.

## Conclusion

In this study, two soybean landraces with stable difference in UFA were screened out from 314 cultivars and landraces. Transcriptomic and phenotype analyses indicated that 40 DAF might be a crucial period for the formation of UFA profiles. The DEGs and pathways affecting UFA metabolism were identified based on the transcriptome data and KEGG database. WGCNA identified six hub genes to be highly correlated with the content of UFAs. Overall, this study provides an important insight into the regulation of fatty acid profiles of soybean seeds and also provides some ideas for the improvement in fatty acid profiles in soybean.

## Data Availability Statement

The original contributions presented in the study are publicly available. This data can be found here: www.ncbi.nlm.nih.gov/bioproject/, PRJNA801060.

## Author Contributions

JL and XW conceived the study and drafted the manuscript. JL, LD, RD, LH, YZ, and LZ performed the experiments. JL analyzed the data. All authors contributed to the article and approved the final version of the manuscript.

## Conflict of Interest

The authors declare that the research was conducted in the absence of any commercial or financial relationships that could be construed as a potential conflict of interest.

## Publisher’s Note

All claims expressed in this article are solely those of the authors and do not necessarily represent those of their affiliated organizations, or those of the publisher, the editors and the reviewers. Any product that may be evaluated in this article, or claim that may be made by its manufacturer, is not guaranteed or endorsed by the publisher.

## Footnotes

1
http://www.r-project.org

## Supplementary Material

Supplementary Figure 1 Global gene expression profiling of HO and LO.

Supplementary Figure 2 Heatmap comparison of the trait-specifical module genes.

Supplementary Table 1 The DEGs at three developmental stages.

Supplementary Table 2 The gene specific primers for qRT-PCR.

Supplementary Table 3 The fatty acid profiles of HO and LO at different stage.

Supplementary Table 4 Gene Ontology analysis of trait-specifical module.

Supplementary Table 5 Kyoto Encyclopedia of Genes and Genomes (KEGG) enrichment analysis of three modules.

Supplementary Table 6 Genes of “plasma membrane” and “monolayer-surrounded lipid storage body.”
