# Supplementary material for: Transcriptomic Analysis Reveals the Regulatory Networks and Hub Genes Controlling the Unsaturated Fatty Acid Contents of Developing Seed in Soybean
Source: Front Plant Sci. 2022 May 12;13:876371. doi: 10.3389/fpls.2022.876371 (PMC9134122; doi:10.3389/fpls.2022.876371)
Supplement: Supplementary Figure 1 — Global gene expression profiling of HO and LO. [file Table_1.DOCX]

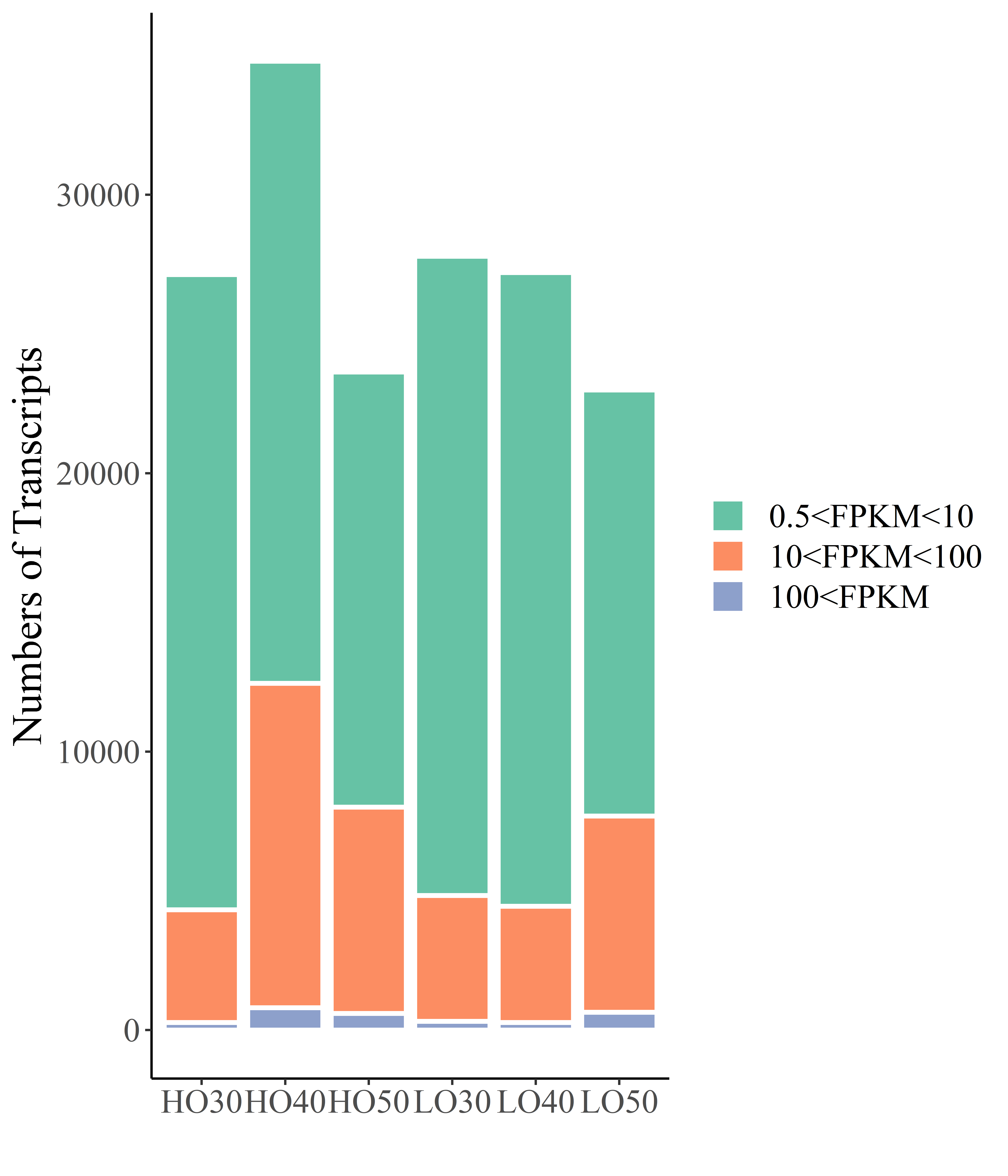


A


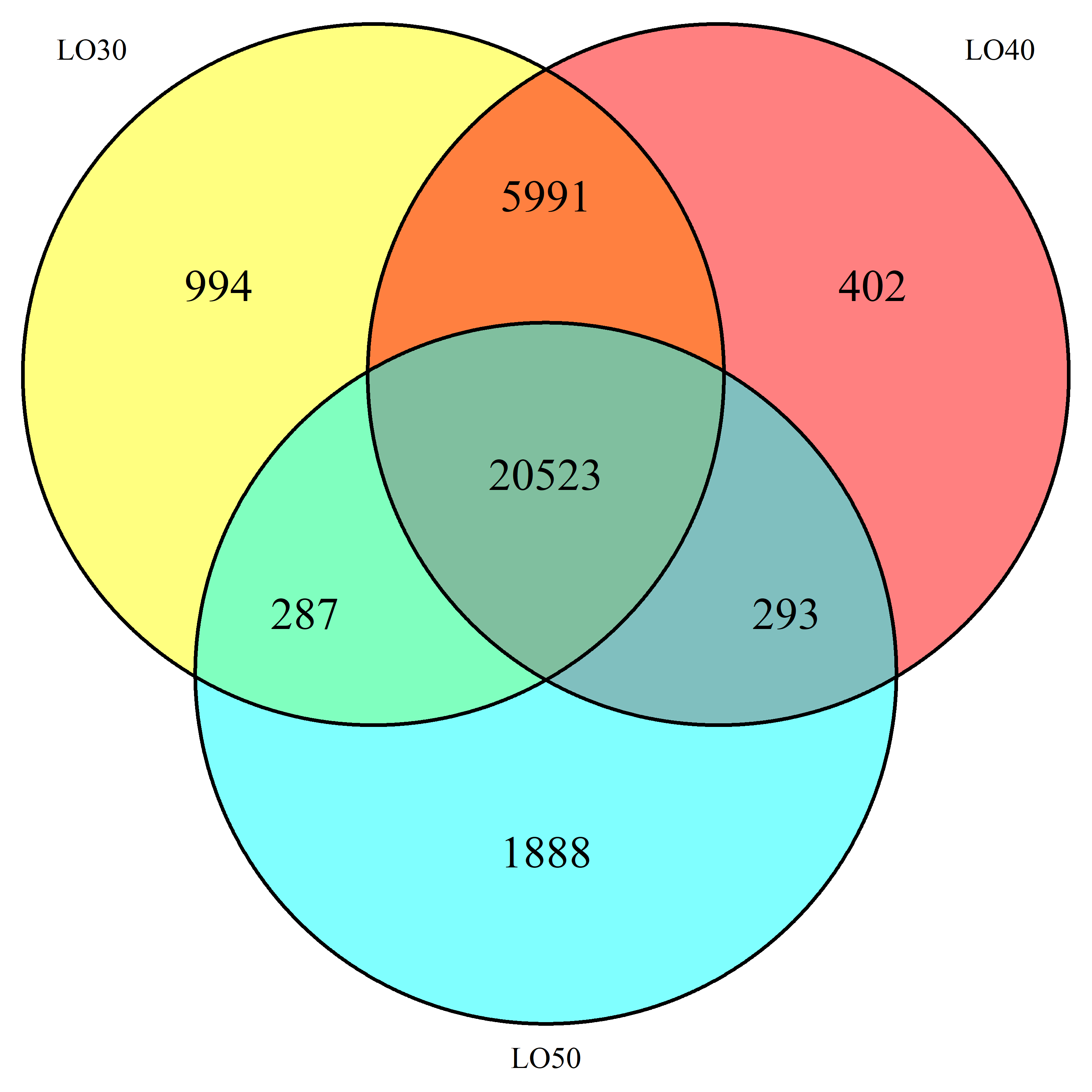


B


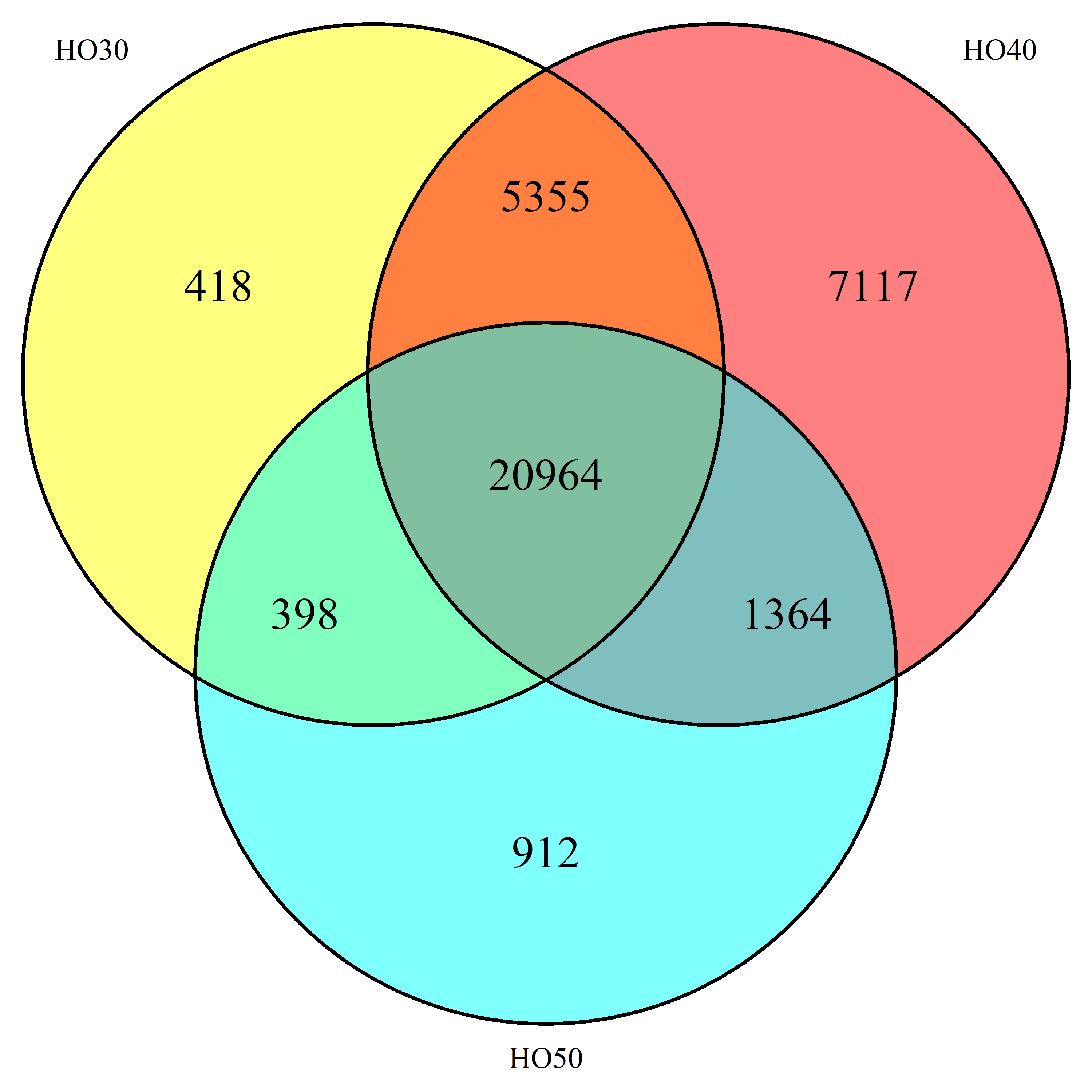


C


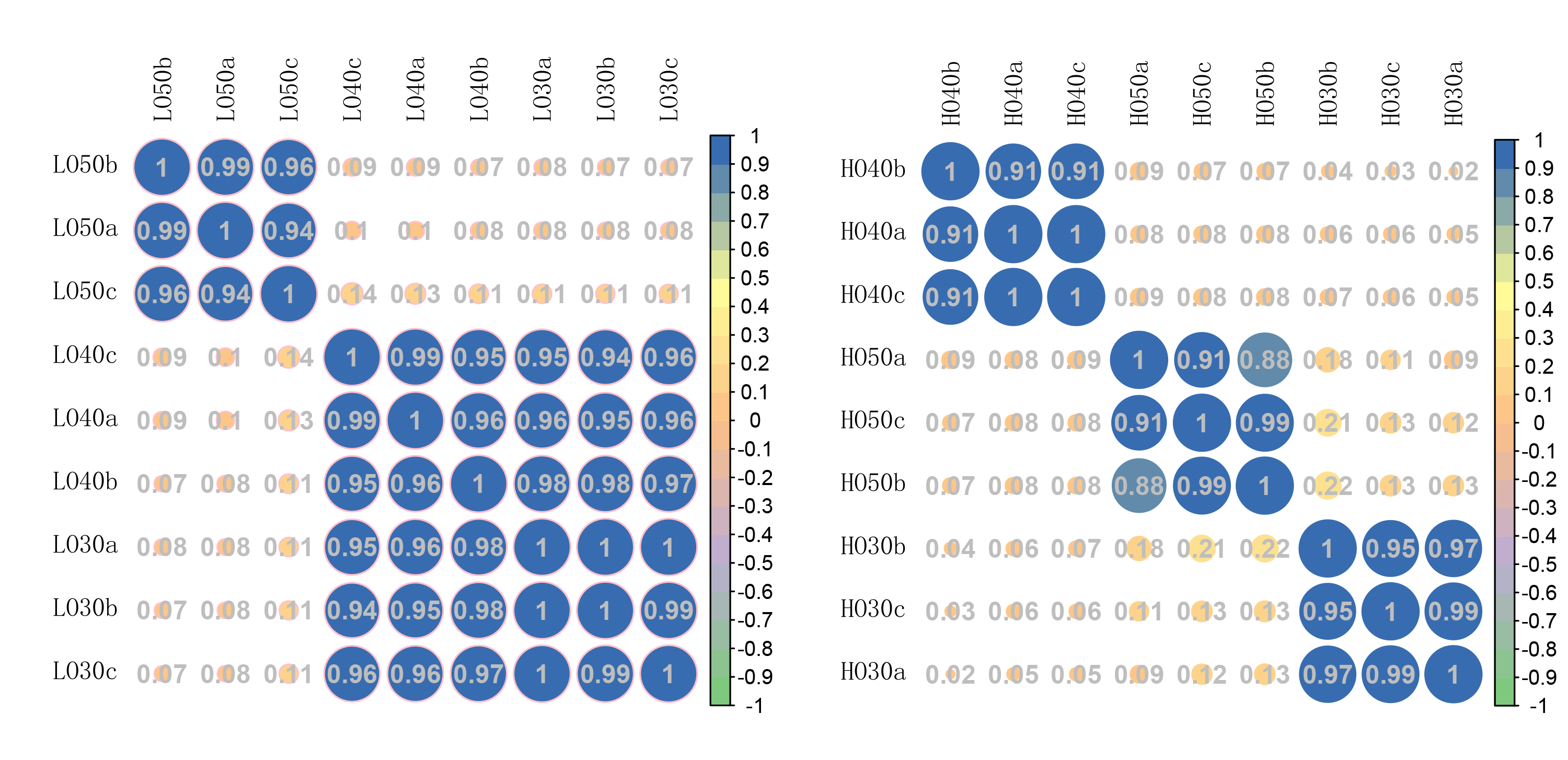


D

**FIGURE S1|** Global gene expression profiling of HO and LO. **(A)**Numbers of detected transcripts in each sample. **(B,C)**Venn diagrams of differentially expressed transcripts among the three samples of **(B)** LO and **(C)** HO. **(D)** Pearson correlation coefficient analysis between biological duplicates.
