# Supplementary material for: Transcriptomic Analysis Reveals the Regulatory Networks and Hub Genes Controlling the Unsaturated Fatty Acid Contents of Developing Seed in Soybean
Source: Front Plant Sci. 2022 May 12;13:876371. doi: 10.3389/fpls.2022.876371 (PMC9134122; doi:10.3389/fpls.2022.876371)
Supplement: Supplementary Figure 2 — Heatmap comparison of the trait-specifical module genes. [file Table_2.DOCX]

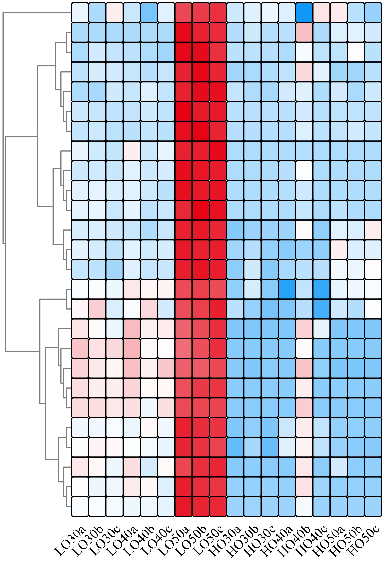

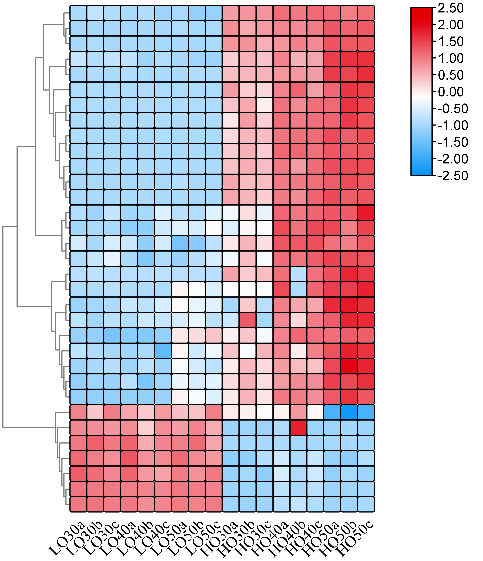

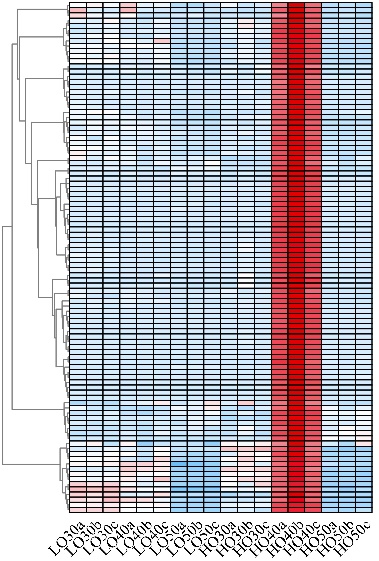


A B C

**FIGURE S2|** Heatmap comparison of the trait-specifical module genes. **(A)**skyblue module; **(B)**purple module; **(C)**yellow module; The heatmap was clustered by pearson method of TBtools software. Heatmap indicate the gene expression level by log2[FPKM] with color scale, each row represents a single gene, the IDs and names of selected DEGs are indicated to the right of the histograms, and each column represents a sample.
